# Association between average screen time and dry eye disease: a secondary analysis of publicly available cross-sectional data

**DOI:** 10.3389/fmed.2026.1942816

**Published:** 2026-09-23

**Authors:** Juan Ling, ChenJia Zhang, LiYing Chen, WenJuan Ma, Li Zhang, Juan Chang

**Affiliations:** 1 Gansu Provincial Hospital, Lanzhou, Gansu Province, China; 2 Clinical College of Chinese Medicine, Gansu University of Chinese Medicine, Lanzhou, Gansu Province, China

**Keywords:** association, asssociation, average screen time, dry eye disease, sectional study

## Abstract

**Background:**

The pervasive use of smartphones and other visual display terminals has heightened concerns regarding ocular health, especially the development or exacerbation of dry eye disease (DED) and inflammatory conjunctivitis. Prolonged screen viewing is consistently linked to a reduced spontaneous blink rate, accelerated tear-film evaporation, and a shortened tear-film break-up time (TBUT), factors that synergistically promote tear-film instability and ocular discomfort.

**Objective:**

This study aimed to investigate the association between excessive daily screen exposure and the prevalence of DED.

**Methods:**

This study is a secondary analysis of a publicly available, de-identified dataset from Kaggle (Dry Eye Disease Dataset, *n* = 20,000 adults aged 18–45 years). We used multivariable logistic regression to examine the association between average screen time and DED, adjusting for demographic, sleep, blood-pressure, body-mass index (BMI), and lifestyle covariates. Generalized additive models (GAMs) with smooth-spline fitting were applied to explore potential non-linear relationships.

**Results:**

Among 20,000 participants, 13,037 (65.2%) were classified as having DED according to the dataset label and 6,963 (34.8%) as not having DED. After full adjustment, each additional hour of average screen time was associated with 2% higher odds of DED [odds ratio [OR] = 1.02, 95% confidence interval [CI] 1.01–1.03, *P* = 0.0015]. This association remained consistent in Model 2 (OR=1.02, 95% CI 1.01–1.03, *P* = 0.0017) and in Model 3 (OR=1.02, 95% CI 1.01–1.03, *P* = 0.0029). Smooth-spline analysis indicated a non-linear dose-response relationship, with a nadir at approximately 3–4 h/day and a sharp increase beyond 9 h/day.

**Conclusions:**

This cross-sectional analysis demonstrates a consistent, dose-dependent association between daily screen exposure and the prevalence of DED. Each additional hour of screen exposure was associated with higher odds of DED.

## 1 Introduction

Dry eye disease (DED) is a multifaceted condition characterized by a loss of tear film homeostasis, leading to ocular discomfort, visual disturbances, and potential damage to the ocular surface. It affects millions worldwide, with prevalence rates varying significantly based on demographics, geographic location, and lifestyle factors. The complexity of DED arises from its multifactorial etiology, which includes environmental factors, systemic diseases (1), and lifestyle choices such as prolonged screen time and digital device usage. In South America, a systematic review revealed a prevalence range of 4% to 77.5%, with a mean prevalence of approximately 39.3% across various populations (2). Notably, higher rates were observed among specific groups such as university students (58.6%) and administrative workers (57.9%). In Africa, a meta-analysis indicated an overall prevalence of 42.0%, with significant variability influenced by factors such as sex and age (3). In Asia, studies have shown that the prevalence of DED is particularly high among specific demographics, including young adults and those engaged in prolonged screen time. Together, these data indicate that DED prevalence is consistently high across world regions but varies with age, sex, occupation, and digital lifestyle, which supports the need for population-specific risk assessment.

The diagnosis of DED is often complicated by the variability of symptoms and the overlap with other ocular conditions. Current diagnostic approaches include subjective assessments through questionnaires and objective evaluations such as tear break-up time (TBUT), osmolarity tests, and ocular surface staining (4, 5).

Recent studies have indicated that DED is increasingly recognized in younger populations, particularly in the context of rising digital device usage among children and adolescents, which has been exacerbated by the global shift towards online learning during the COVID-19 pandemic. This trend necessitates a deeper understanding of the condition's prevalence, risk factors, and the impact of screen time on ocular health, especially in younger individuals (6, 7). For example, prolonged screen use has been associated with ocular discomfort, burning sensations, dryness, and visual fluctuations (8–10). The mechanisms underlying this association may include altered blinking patterns and increased tear evaporation due to reduced blink frequency during screen use (11–13). Furthermore, the digital lifestyle, characterized by extended hours of exposure to screens, has been linked to changes in tear film dynamics, contributing to the development of dry eye disease (9, 10).

DED diminishes visual comfort, occupational performance, and overall well-being, and has also been linked to heightened psychological distress and reduced academic achievement (14–17). The cumulative expenses involved in the prevention, diagnosis, and management of DED therefore place a considerable economic burden on healthcare systems and affected individuals (18, 19). Because spontaneous blinking adapts to the demands of specific visual tasks, the magnitude of screen-related DED manifestations varies accordingly (11, 12). Young adults with digital eye strain frequently display a symptom profile that parallels DED, including ocular discomfort, suppressed blink rate, and aggravated eye symptoms during screen viewing, all of which can erode productivity and quality of life (9). This cross-sectional investigation is designed to refine the current understanding of the association between screen exposure and DED, generating evidence that can inform clinical decision-making and steer the development of future therapeutic strategies.

Although the association between screen time and DED has been studied for many years, most previous studies have focused on specific occupational groups, children, or relatively small samples. The present study contributes new information by analysing a large, demographically broad sample of 20,000 adults aged 18–45 years; by modelling screen time as a continuous exposure with spline-based dose-response curves; by adjusting for a broad set of demographic, sleep, and lifestyle covariates; and by identifying sex, age, BMI, and caffeine-related subgroups in which the association is stronger. These features move beyond simple replication and provide a quantitative basis for targeted screen-time counselling.

Despite this background, important uncertainties remain: few previous studies quantified the dose-response shape across the full screen-time range, adjusted simultaneously for sleep, blood pressure, BMI, and lifestyle factors, or identified which demographic subgroups carry the highest risk. These gaps justify the present secondary analysis of a large, publicly available, de-identified dataset.

## 2 Methods

### 2.1 Study population

This secondary analysis used the ‘Dry Eye Disease Dataset' from the open-access Kaggle repository (https://www.kaggle.com/datasets/dakshnagra/dry-eye-disease/code). The dataset contains de-identified records of 20,000 adults aged 18–45 years. The outcome variable, DED, was the binary dry eye disease indicator included in the dataset; the dataset page does not provide a detailed clinical diagnostic protocol. We therefore analysed the DED label as reported by the original data collector and explicitly address this limitation in the Discussion.

The dataset file does not report the original study setting, data-collection period, or eligibility criteria. Because these details are not available in the public archive, they cannot be reconstructed from the dataset and are acknowledged as limitations.

### 2.2 Data source and dataset screening

To select a suitable open dataset, we searched Kaggle for publicly available dry eye datasets on 22 August 2026. Candidate datasets were screened using predefined criteria: (1) individual-level records rather than aggregated or image-only data; (2) inclusion of a screen-time variable; (3) availability of demographic, sleep, and lifestyle covariates; (4) de-identified and publicly downloadable content; and (5) a sample size sufficient for stratified dose-response analysis. The ‘Dry Eye Disease Dataset' uploaded by Daksh Nagra met all criteria and was downloaded from https://www.kaggle.com/datasets/dakshnagra/dry-eye-disease/code (also accessible at https://www.kaggle.com/datasets/dakshnagra/dry-eye-disease).

Before analysis, we verified the downloaded file (Dry_Eye_Dataset.csv). It contains 20,000 rows and 26 columns, ages ranging from 18 to 45 years, complete values for all variables, and no direct personal identifiers. Because PubMed indexes peer-reviewed publications rather than raw data repositories, we used PubMed-indexed literature to define and cross-check the epidemiological and biological plausibility of the variables, and we used the Kaggle dataset as the de-identified individual-level source for the present secondary analysis.

The original data-collection tools, validation procedures, and eligibility criteria are not documented in the Kaggle archive, and no parent publication was identified. We therefore describe the variables as recorded and explicitly acknowledge this methodological limitation.

### 2.3 Ethics statement

The present study is a secondary analysis of a fully de-identified, publicly available dataset. Because it involved no new recruitment, intervention, or access to identifiable participant information, additional ethical approval was not required. The dataset page does not provide verifiable documentation of the original informed-consent process; we therefore do not assert that participant-level consent documentation is available in the dataset.

### 2.4 Variables

Demographic and clinical information, including age, sex, average screen time (hours per day), sleep duration, blood pressure, body-mass index (BMI), sleep disorders, and other lifestyle factors, was extracted from the downloaded dataset. Height and weight were used to calculate BMI as weight (kg) divided by height squared (m squared). Average screen time was the self-reported average daily hours of screen use recorded in the dataset. Categorical variables, including sleep disorder, wake up during night, feel sleepy during day, caffeine consumption, alcohol consumption, smoking, medical issue, ongoing medication, smart device before bed, discomfort eye strain, redness in eye, and itchiness or irritation in eye, were analysed as reported.

Height and weight were recorded in the dataset; the original measurement instruments are not documented in the public archive. The original measurement instrument for average screen time is not documented in the public archive. The categorical lifestyle variables were recorded in the Kaggle dataset as self-reported measures; because the original questionnaire is not publicly available, we cannot describe the exact instrument or validation procedure.

### 2.5 Statistical analysis

Continuous variables were initially assessed for normality using the Shapiro–Wilk and Kolmogorov–Smirnov tests, and homogeneity of variance was evaluated. Variables that met these assumptions were compared among groups using one-way analysis of variance (ANOVA); otherwise, non-parametric comparisons were conducted using the Kruskal–Wallis test (20, 21).

In this study, categorical data were summarized using frequencies and proportions, while continuous data were described using means ± standard deviations (SD). The association between DED and average screen time was examined using multivariable logistic regression with smooth curve fitting, while accounting for pertinent clinical covariates. Generalized additive models (GAMs) with smooth splines were applied to explore potential non-linear relationships between screen time and DED. Subgroup analyses and interaction tests were also conducted to determine whether the association varied across different strata of interest.

The presence of multicollinearity among independent variables was evaluated using the variance inflation factor (VIF). Variables with a VIF of 5 or higher were deemed to exhibit high collinearity and were subsequently excluded from the model (22, 23). This approach ensured that only the most relevant variables were retained, thereby improving the stability and interpretability of the model. All statistical analyses were conducted using EmpowerStats, version 4.1 (www.empowerstats.net; X&Y Solutions, Inc., Boston, MA), and the R software environment, version 4.2.0 (The R Foundation; The R Project for Statistical Computing).

## 3 Results

A total of 20,000 participants were included in the study, comprising 13,037 (65.2%) individuals classified as having DED and 6,963 (34.8%) classified as not having DED according to the dataset label. The baseline characteristics of the participants are summarized in Table 1.

**Table 1:** Baseline characteristics of the study participants (*n* = 20,000).

Characteristic	Dry eye disease (*N* = 13,037)	No dry eye disease (*N* = 6,963)	Standardize diff.	*P*—value
Age(years)	31.5 ± 8.1	31.4 ± 8.0	0.0 (−0.0, 0.0)	0.457
Sleep Duration(hours)	7.0 ± 1.7	7.0 ± 1.7	0.0 (−0.0, 0.0)	0.902
Systolic Blood Pressure(mmHg)	115.1 ± 14.8	114.8 ± 14.7	0.0 (−0.0, 0.0)	0.303
Diastolic Blood Pressure(mmHg)	75.0 ± 9.0	75.1 ± 8.9	0.0 (−0.0, 0.0)	0.475
BMI(kg/m^2^)	25.1 ± 6.6	25.0 ± 6.5	0.0 (−0.0, 0.0)	0.308
Gender			0.0 (0.0, 0.1)	0.021
Female	6,578 (50.5%)	3,394 (48.7%)		
Male	6,459 (49.5%)	3,569 (51.3%)		
Sleep Disorder			0.0 (−0.0, 0.0)	0.711
No	6,551 (50.2%)	3,518 (50.5%)		
Yes	6,486 (49.8%)	3,445 (49.5%)		
Wake Up During Night			0.0 (−0.0, 0.0)	0.964
No	6,520 (50.0%)	3,480 (50.0%)		
Yes	6,517 (50.0%)	3,483 (50.0%)		
Feel Sleepy During Day			0.0 (−0.0, 0.0)	0.504
No	6,612 (50.7%)	3,566 (51.2%)		
Yes	6,425 (49.3%)	3,397 (48.8%)		
Caffeine Consumption			0.0 (−0.0, 0.0)	0.689
No	6,447 (49.5%)	3,464 (49.7%)		
Yes	6,590 (50.5%)	3,499 (50.3%)		
Alcohol Consumption			0.0 (−0.0, 0.1)	0.111
No	6,459 (49.5%)	3,532 (50.7%)		
Yes	6,578 (50.5%)	3,431 (49.3%)		
Smoking			0.0 (−0.0, 0.0)	0.469
No	6,554 (50.3%)	3,463 (49.7%)		
Yes	6,483 (49.7%)	3,500 (50.3%)		
Medical Issue			0.0 (−0.0, 0.0)	0.879
No	6,596 (50.6%)	3,515 (50.5%)		
Yes	6,441 (49.4%)	3,448 (49.5%)		
Ongoing Medication			0.0 (−0.0, 0.0)	0.554
No	6,485 (49.7%)	3,433 (49.3%)		
Yes	6,552 (50.3%)	3,530 (50.7%)		
Smart Device Before Bed			0.0 (−0.0, 0.1)	0.06
No	6,580 (50.5%)	3,417 (49.1%)		
Yes	6,457 (49.5%)	3,546 (50.9%)		
Discomfort Eye Strain			0.2 (0.2, 0.3)	<0.001
No	5,971 (45.8%)	3,992 (57.3%)		
Yes	7,066 (54.2%)	2,971 (42.7%)		
Redness In Eye			0.2 (0.2, 0.2)	<0.001
No	6,110 (46.9%)	4,019 (57.7%)		
Yes	6,927 (53.1%)	2,944 (42.3%)		
Itchiness/Irritation In Eye			0.2 (0.2, 0.2)	<0.001
No	6,077 (46.6%)	3,986 (57.2%)		
Yes	6,960 (53.4%)	2,977 (42.%)8		

Mean ± SD for continuous variables; *P* value was calculated by weighted linear regression model. % for categorical variables; *P* value was calculated by weighted chi-square test.

The association between average screen time and DED was analysed using three models with increasing levels of adjustment for potential confounders (Table 2). In Model 1 (unadjusted), each additional hour of average screen time was associated with 2% higher odds of DED (OR=1.02, 95% CI: 1.01–1.03, *P* = 0.0015). This association remained consistent after adjusting for sex and age in Model 2 (OR=1.02, 95% CI: 1.01–1.03, *P* = 0.0017) and after further adjustment for multiple covariates, including sleep duration, blood pressure, BMI, sleep disorders, and other lifestyle factors in Model 3 (OR=1.02, 95% CI: 1.01–1.03, *P* = 0.0029).

**Table 2:** Association between average screen time and DED.

Average screen time (hours)	Model 1 OR (95% CI)	Model 2 OR (95% CI)	Model 3 OR (95% CI)
Overall	1.02 (1.01, 1.03) 0.0015	1.02 (1.01, 1.03) 0.0017	1.02 (1.01, 1.03) 0.0029
Average screen time (hours) tertile			
Low	1.0	1.0	1.0
Middle	0.99 (0.92, 1.06) 0.7772	0.99 (0.92, 1.06) 0.7802	0.99 (0.92, 1.07) 0.8395
High	1.10 (1.03, 1.18) 0.0074	1.10 (1.03, 1.18) 0.0082	1.09 (1.02, 1.18) 0.0149

Model 1, no covariates were adjusted. Model 2: sex and age were adjusted. Model 2: Sex and age were adjusted. Model 3: Sex, age, sleep duration, systolic blood pressure, diastolic blood pressure, BMI, sleep disorder, wake up during night, feel sleepy during day, caffeine consumption, alcohol consumption, smoking, medical issue, ongoing medication, smart device before bed, discomfort eye strain, redness in eye, and itchiness/irritation in eye were adjusted.

When average screen time was categorized into tertiles (low, middle, high), the highest tertile was associated with 10% higher odds of DED compared with the lowest tertile in Model 1 (OR=1.10, 95% CI: 1.03–1.18, *P* = 0.0074) and Model 2 (OR=1.10, 95% CI: 1.03–1.18, *P* = 0.0082). This association was slightly attenuated but remained significant in Model 3 (OR=1.09, 95% CI: 1.02–1.18, *P* = 0.0149). The middle tertile was not significantly associated with DED in any of the models (OR=0.99, *P* > 0.05).

These findings suggest that higher average screen time is independently associated with increased odds of dry eye disease, even after accounting for multiple potential confounding factors.

Subgroup analyses and interaction tests were performed to further examine the association between average screen time and DED and to evaluate potential factors that may influence this association (Table 3). Subgroups were categorized based on sex (male or female), sleep disorder, wake up during night, feel sleepy during day, caffeine consumption, alcohol consumption, smoking, medical issue, ongoing medication, smart device before bed, age (18–26 years, 27–35 years, and 36–45 years), systolic blood pressure (90.0–106.0 mmHg, 107.0–123.0 mmHg, and 124.0–140.0 mmHg), diastolic blood pressure (60.0–69.0 mmHg, 70.0–79.0 mmHg, and 80.0–90.0 mmHg), and BMI (12.5–21.5 kg/m2, 21.5–27.4 kg/m2, and 27.4–44.4 kg/m2). The *P* value for interaction of sex was 0.0293, and no significant interactions were detected in the other subgroups (*P* > 0.05).

**Table 3:** Association between average screen time and dry eye disease, stratified by factors.

Characteristic	No of participants	OR (95% CI)	*P* value	*P* for interaction
Stratified by Gender				0.0293
male	10,028	1.1 (0.9, 1.2)	0.508	
female	9,972	1.4 (1.2, 1.6)	<0.001	
Stratified by sleep disorder				0.7448
No	10,069	1.2 (1.0, 1.4)	0.013	
Yes	9,931	1.2 (1.0, 1.4)	0.043	
Stratified by wake up during night				0.9049
No	10,000	1.2 (1.0, 1.4)	0.02	
Yes	10,000	1.2 (1.0, 1.4)	0.03	
Stratified by feel sleepy during day				0.1347
No	10,178	1.1 (0.9, 1.3)	0.218	
Yes	9,822	1.3 (1.1, 1.5)	0.001	
Stratified by caffeine consumption				
No	9,911	1.1 (0.9, 1.2)	0.463	
Yes	10,089	1.4 (1.2, 1.6)	<0.001	
Stratified by alcohol consumption				0.1335
No	9,991	1.2 (1.0, 1.4)	0.072	
Yes	10,009	1.2 (1.1, 1.5)	0.006	
Stratified by smoking				0.3377
No	10,017	1.3 (1.1, 1.5)	0.004	
Yes	9,983	1.1 (1.0, 1.3)	0.113	
Stratified by medical issue				0.2467
No	10,111	1.1 (1.0, 1.3)	0.147	
Yes	9,889	1.3 (1.1, 1.5)	0.002	
Stratified by ongoing medication				0.8726
No	9,918	1.2 (1.0, 1.4)	0.02	
Yes	10,082	1.2 (1.0, 1.4)	0.031	
Stratified by smart device before bed				0.8802
No	9,997	1.2 (1.0, 1.4)	0.032	
Yes	10,003	1.2 (1.0, 1.4)	0.018	
Stratified by age tertile				0.3,565
Low (18–26 years)	6,476	1.2 (1.0, 1.5)	0.073	
Middle (27–35 years)	6,483	1.4 (1.1, 1.7)	0.002	
High (36–45 years)	7,041	1.1 (0.9, 1.3)	0.518	
Stratified by systolic blood pressure tertile				0.5,807
Low (90.0–106.0 mmHg)	6,663	1.2 (1.0, 1.4)	0.104	
Middle (107.0–123.0 mmHg)	6,654	1.3 (1.1, 1.6)	0.008	
High (124.0–140.0 mmHg)	6,683	1.1 (0.9, 1.4)	0.211	
Stratified by diastolic blood pressure tertile				0.5053
Low (60.0–69.0 mmHg)	6,446	1.1 (0.9, 1.3)	0.396	
Middle (70.0–79.0 mmHg)	6,402	1.2 (1.0, 1.5)	0.04	
High (80.0–90.0 mmHg)	7,152	1.3 (1.1, 1.5)	0.011	
Stratified by BMI tertile				0.9062
Low (12.5–21.5 kg/m^2^)	6,660	1.2 (1.0, 1.4)	0.141	
Middle (21.5–27.4 kg/m^2^)	6,672	1.2 (1.0, 1.5)	0.041	
High (27.4–44.4 kg/m^2^)	6,668	1.2 (1.0, 1.5)	0.048	

Subgroup analyses stratified by sex, sleep disorder, wake up during night, feel sleepy during day, caffeine consumption, alcohol consumption, smoking, medical issue, ongoing medication, smart device before bed, age, systolic blood pressure, diastolic blood pressure, and BMI.

In the overall sample, smooth-spline fitting showed a non-linear dose-response pattern. The predicted probability of DED declined slightly at low screen exposure, reached a nadir (∼0.64) at approximately 3–4 h/day, and then rose steadily, with a sharp upturn beyond 9 h/day and a peak near 0.70 (Figure 1). Stratification by BMI tertiles revealed upward displacement of the exposure-response curves in the middle and high BMI categories relative to the low BMI reference; the high-BMI group consistently exhibited the greatest risk, with a sharp escalation (∼0.70) at 9–10 h/day (Figure 2). Age-stratified curves diverged notably: the youngest tertile (18–26 years) showed a modest linear increase, whereas the middle tertile (27–35 years) displayed a U-shaped trajectory, culminating in the highest risk (∼0.74) at 9 h/day or more (Figure 3). In sex-stratified analyses, the male curve climbed steadily from baseline, reaching ∼0.68 at 10 h/day, whereas the female curve declined between 2 and 6 h/day before ascending sharply and intersecting the male trajectory beyond 9 h/day, peaking at ∼0.70 (Figure 4). Generalized additive models (GAMs) with smooth splines were used to estimate the dose-response association between average screen time and DED. The *P* value for non-linearity in Figures 1–4 represents the likelihood ratio test for threshold effects. In every panel, the red line represents the fitted smooth curve and the blue shaded band represents the 95% confidence interval. Figure 1 covariates adjusted: sex, sleep disorder, wake up during night, feel sleepy during day, caffeine consumption, alcohol consumption, smoking, medical issue, ongoing medication, smart device before bed, age, systolic blood pressure, diastolic blood pressure, BMI, discomfort eye strain, redness in eye, and itchiness or irritation in eye. Figure 2 covariates adjusted: all of the above except BMI. Figure 3 covariates adjusted: all of the above except age. Figure 4 covariates adjusted: all of the above except sex.

**Figure 1:**
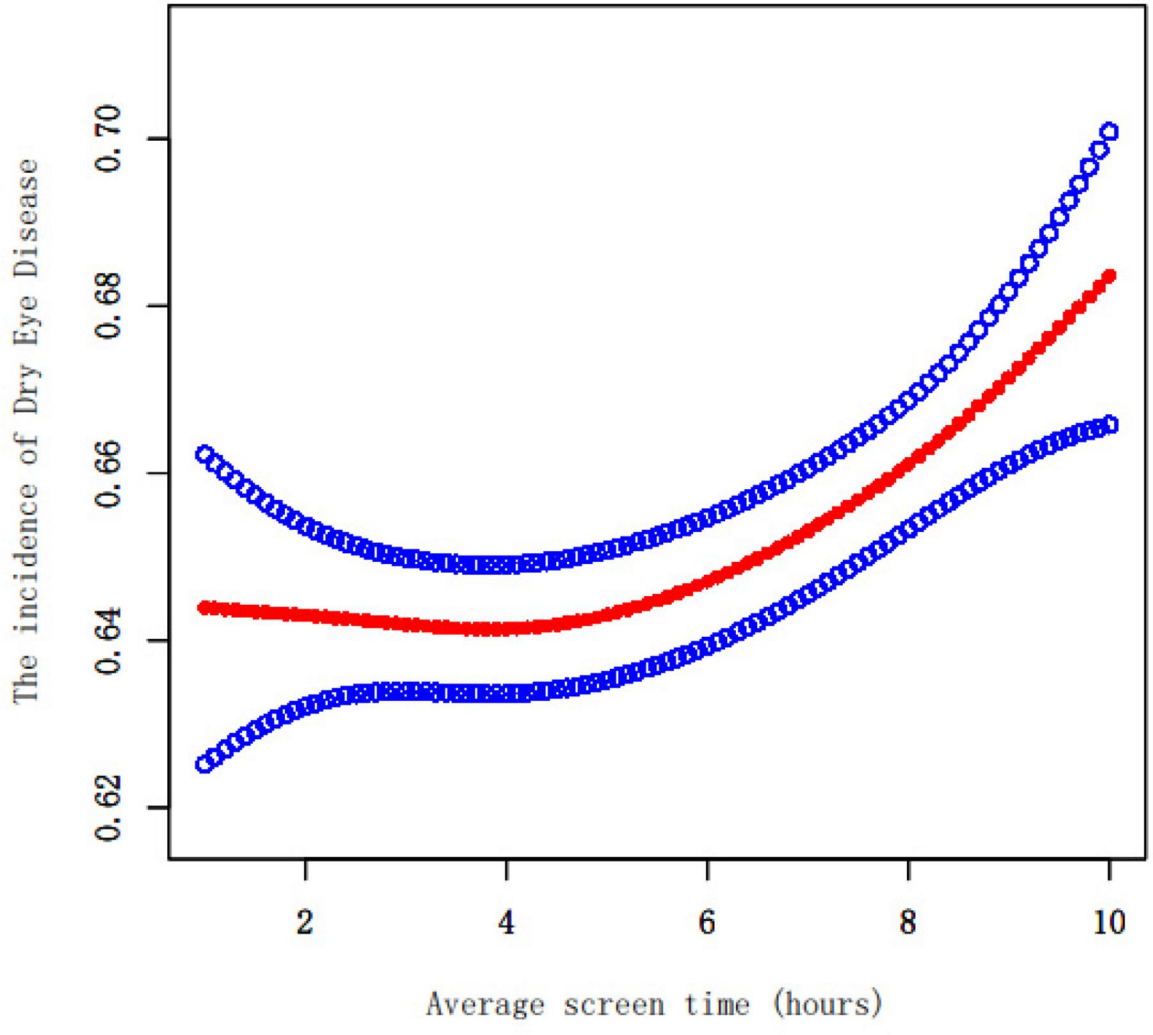
Association between average screen time and DED. Red line, fitted smooth curve; blue shaded band, 95% confidence interval.

**Figure 2:**
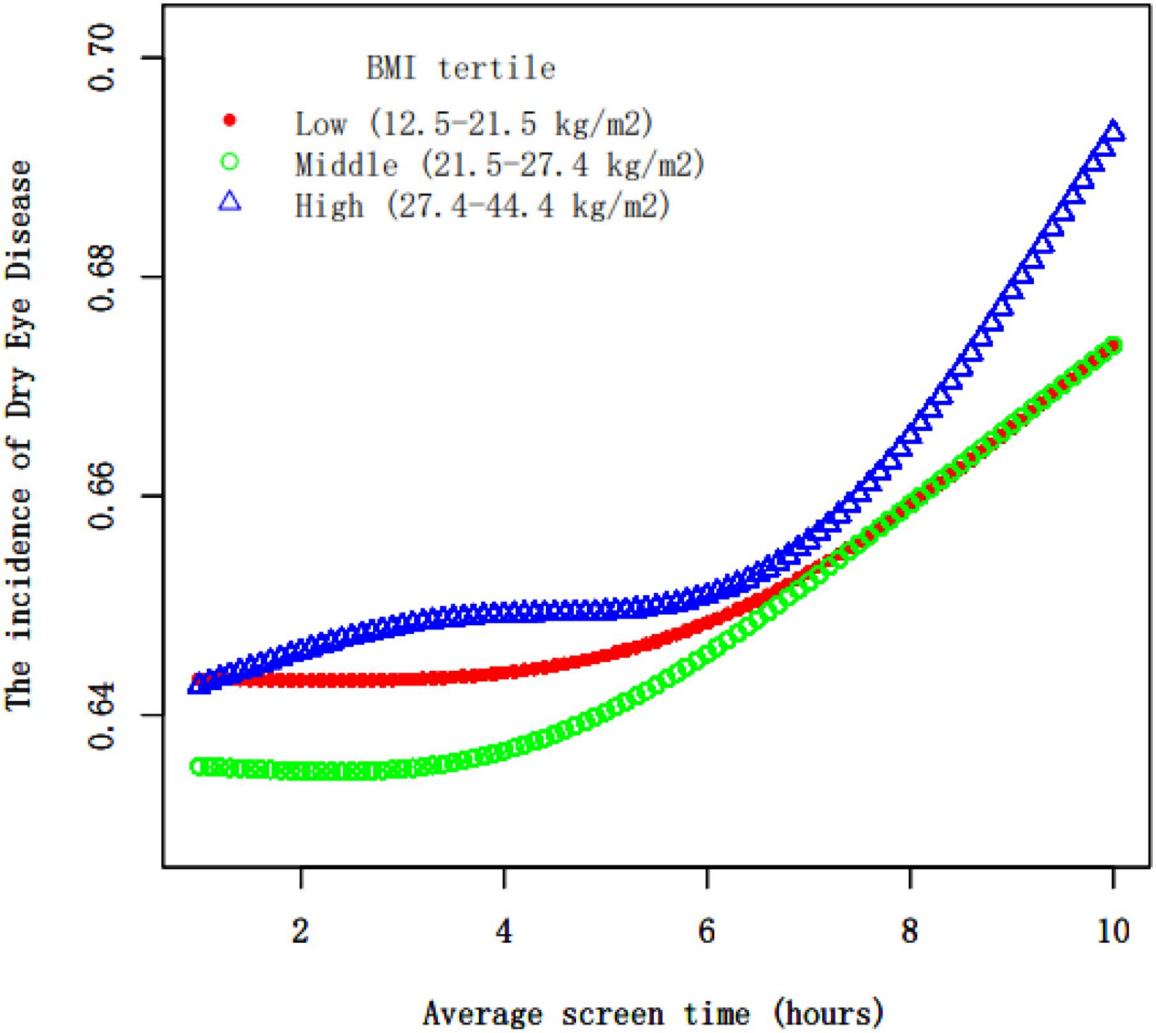
Subgroup analysis stratified by BMI. Red line, fitted smooth curve; blue shaded band, 95% confidence interval.

**Figure 3:**
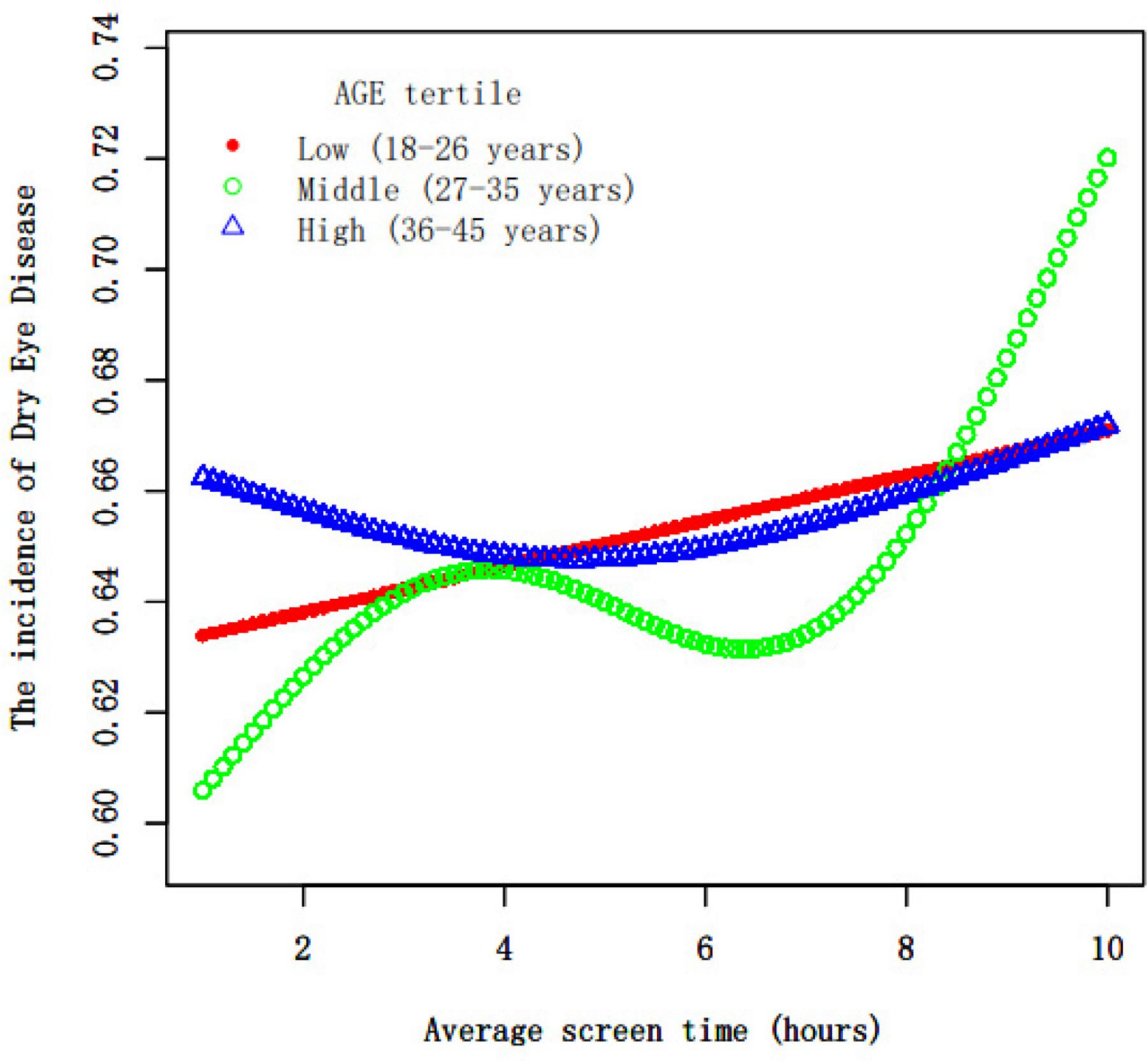
Subgroup analysis stratified by age. Red line, fitted smooth curve; blue shaded band, 95% confidence interval.

**Figure 4:**
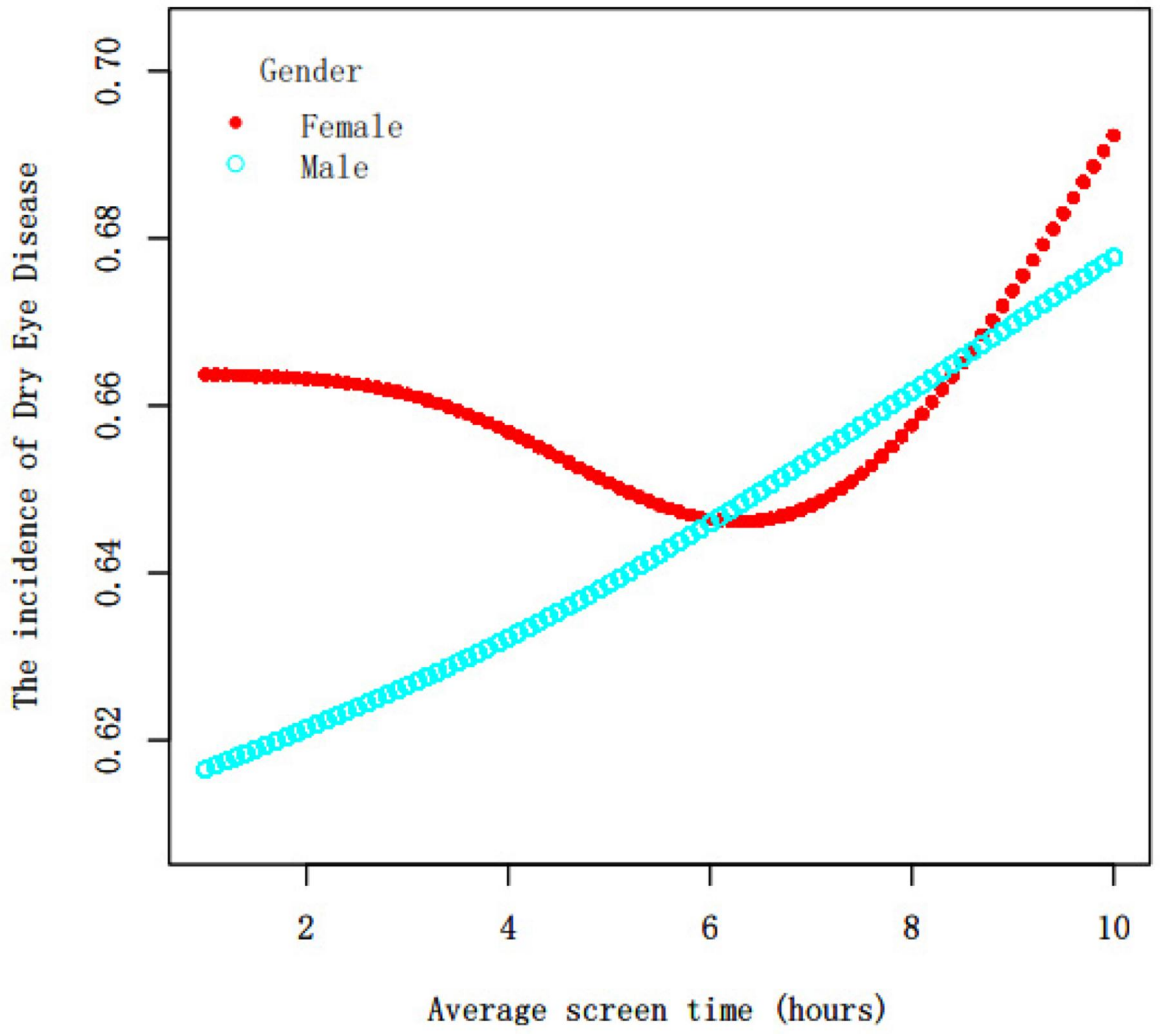
Subgroup analysis stratified by gender. Red line, fitted smooth curve; blue shaded band, 95% confidence interval.

## 4 Discussion

In this cross-sectional study of 20,000 adults aged 18–45 years, we examined the association between average daily screen time and DED. The positive association persisted across multivariable models and was strongest at high screen-exposure levels and in specific subgroups, including women, adults aged 27–35 years, and participants with higher BMI or regular caffeine consumption. In the tertile analysis, the association was observed in the highest screen-time tertile relative to the lowest, consistent with the continuous dose-response pattern. These results are presented in detail in Tables 2, 3 and Figures 1–4.

Beyond replicating the established screen-time-DED association, the present study contributes new information in three ways. First, it provides a continuous, spline-based dose-response curve across the full range of screen exposure in a large adult sample. Second, it simultaneously adjusts for a broad set of demographic, sleep, and lifestyle covariates. Third, it identifies clinically relevant high-risk subgroups, including women, adults aged 27–35 years, and participants with higher BMI or regular caffeine consumption, thereby providing clear targets for preventive counselling.

Our findings are consistent with previous studies, although those studies were smaller or restricted to specific populations. In a cross-sectional study of 1,025 Japanese office workers, Uchino et al. (24) reported higher odds of DED with each additional hour of monitor use (adjusted OR about 1.08), and Moon et al. (25) found that more than 4 h/day of screen time increased DED risk among 1,460 Korean schoolchildren (OR 1.73). The larger, more heterogeneous sample and spline-based dose-response modelling in our study complement these findings by providing a continuous exposure-response curve across a broad adult age range. The null finding in 300 medical students reported by Logaraj et al. (26) may reflect their smaller sample, questionnaire-only case definition, and limited adjustment. Mechanistically, reduced blinking accelerates tear-film evaporation (13), while high-energy blue light from screens may alter tear osmolarity and trigger ocular surface inflammation (10). Although we did not directly measure these mechanisms, they are consistent with the higher DED odds observed at prolonged screen times (10, 13) and may be more pronounced in adults with high cumulative exposure.

Clinically, these findings suggest that daily screen duration, usage context, and sex-related susceptibility should be considered in DED assessment, together with tear-film diagnostics, to support earlier case finding. Targeted counselling and preventive measures are particularly relevant for high-risk strata such as women, adults aged 27–35 years, and heavy caffeine users. At the policy level, consistent with Moon et al. (25), who reported increased risk with more than 4 h/day of screen use, our findings support considering a screen-time benchmark in occupational health and school vision guidelines; however, a definitive threshold cannot be established from this cross-sectional analysis. Employers and educators may also be encouraged to promote regular screen breaks and ergonomic monitor settings (9, 10).

### 4.1 Strengths and limitations

This study has several limitations. First, its observational, cross-sectional design allows detection of associations but precludes causal inference. Second, unmeasured confounding cannot be fully excluded. Third, the dataset was obtained from a public repository rather than a formal population-based survey; the DED label was provided by the original data collector, and no standardized diagnostic protocol is documented, so outcome misclassification is possible. Fourth, the de-identified dataset does not provide verifiable information about the original sampling frame, data-collection procedures, or informed-consent process. Fifth, screen time and lifestyle variables were self-reported, which may introduce recall bias. Finally, although the sample is large, it may not be representative of all regions or clinical populations.

This study has several strengths, including a large sample of 20,000 adults, a broad age range, adjustment for a broad set of demographic, sleep, blood-pressure, BMI, and lifestyle covariates, and spline-based dose-response modelling. These features add new information beyond smaller or occupation-specific studies. At the same time, the limitations described above, especially the absence of a documented diagnostic protocol, the unavailability of the original data-collection instruments, and the cross-sectional design, should be considered when interpreting the results.

## 5 Conclusion

Our findings establish a consistent, dose-dependent association between daily screen exposure and the prevalence of DED. Each additional hour of screen time was associated with higher odds of DED, independent of the recorded demographic, sleep, blood-pressure, BMI, and lifestyle covariates. These results highlight the clinical value of integrating detailed screen-time assessments into routine ophthalmic evaluations and DED risk stratification. Such behavioural metrics could facilitate earlier identification of high-risk individuals and inform targeted preventive strategies, including patient education, ergonomic adjustments, and environmental modifications. Future longitudinal and interventional studies are warranted to confirm causality, elucidate underlying mechanisms, and evaluate the effectiveness of tailored screen-time reduction programs in reducing the DED burden across diverse populations.

## Data Availability

The datasets presented in this study can be found in online repositories. The names of the repository/repositories and accession number(s) can be found in the article/Supplementary Material.
